# Supplementary material for: Mining the Species Diversity of Lacewings: New Species of the Pleasing Lacewing Genus Dilar Rambur, 1838 (Neuroptera, Dilaridae) from the Oriental Region
Source: Insects. 2021 May 14;12(5):451. doi: 10.3390/insects12050451 (PMC8157007; doi:10.3390/insects12050451)
Supplement: Supplementary file 1 [file insects-12-00451-s001.zip › Supplementary file 1_Abbreviation of Collections for XML.pdf]

insects

Supplementary File S1. Full Names for the Abbreviations of Collections in Supplementary Table S1.

CASC: Entomological Collection of California Academy of Sciences, San Francisco, CA, U.S.A.
CAU: Entomological Museum, China Agricultural University, Beijing, China
CHRR: Collection of Hubert and Renate Rausch, Scheibbs, Austria
CSCA: California Department of Food and Agriculture, the California State Collection of Arthropods, Sacramento,
U.S.A.
FARU: Faculty of Agriculture of Razi University, Kermanshah, Iran
FSCA: Florida State Collection of Arthropods, Florida, U.S.A.
HUAC: Collection of H. Aspöck & U. Aspöck, Vienna, Austria
ISNB: Institut Royal des Sciences Naturelles de Belgique, Brussels, Belgium
IZCAS: Institute of Zoology, Chinese Academy of Sciences, Beijing, China
MCZ: Museum of Comparative Zoology, Cambridge, MA, USA
MB: Museum of Natural Sciences of Barcelona, Barcelona, Spain
MFN: Museum für Naturkunde, Berlin, Germany
MG: Museo Civico di Storia Naturale G. Doria, Génova, Italy
MNCNM: Museo Nacional de Ciencias Naturales, Madrid, Spain
MNHN: Muséum National d'Histoire Naturelle, Paris, France
MNM: Magyar Nemzeti Múzeum, Budapest, Hungary
MZUN: Museo di Zoologia, Università di Napoli, Napoli, Italy
NHM: Natural History Museum, London, U.K.
NHMB: Naturhistorisches Museum Basel, Basel, Switzerland
NHMG: Natural History Museum Geneva, Geneva, Switzerland
NIAES: National Institute for Agro-Environmental Sciences, Tsukuba, Japan
NMNH: National Museum of Natural History, Smithsonian Institutions, Washington,
D.C., U.S.A.
NMP: National Museum der Naturwissenschaft Entomologische Abteilung, Prague, Czech Republic.
NMS: National Museum of Scotland, Edinburgh, Scotland
NMW: Natural History Museum, Vienna, Austria
NSMT: National Museum of Nature and Science, Tokyo, Japan
PCWH: Collection of William R. Bert Hynd, Farnham, Surrey, U.K.
PPRIP: Plant Protection Research Institute, Pretoria, South Africa
SEHU: Entomological Collection of Hokkaido University Museum, Sapporo, Japan
TAMUIC: Texas A&M University Insect Collection, Texas, U.S.A
UAB: Universidad Autonoma, Bellaterra, Barcelona, Spain
UAH: University of Alcalá, Madrid, Spain
UCD: University of California, Davis, California, U.S.A
VM: Collection of Monserrat, Universidad Complutense, Madrid, Spain
ZMUM: Zoological Museum, Moscow State, University, Moscow, Russia
ZSM: Zoologische Staatssammlung München, Munich, Germany
