# Supplementary material for: Mining the Species Diversity of Lacewings: New Species of the Pleasing Lacewing Genus Dilar Rambur, 1838 (Neuroptera, Dilaridae) from the Oriental Region
Source: Insects. 2021 May 14;12(5):451. doi: 10.3390/insects12050451 (PMC8157007; doi:10.3390/insects12050451)
Supplement: Supplementary file 1 [file insects-12-00451-s001.zip › Supplementary file 2_References for XML.pdf]

insects MDPI

Supplementary File S2. References with Source Data of Distribution Records Herein Summarized.

1. Aspöck, H. & Aspöck, U. Neue Dilariden aus Asien (Neuroptera, Planipennia). *Entomologisches Nachrichtenblatt, Wien* **1967**, 14, 56–59.
2. Aspöck, H. & Aspöck, U. Zwei weitere neue Spezies des Genus *Dilar* Rambur (Neuroptera, Planipennia) aus Asien. (Vorläufige Mitteilung). *Entomologisches Nachrichtenblatt, Wien* **1968**, 15, 3–6.
3. Aspöck, H., Aspöck, U. & Hölzel, H. *Die Neuropteren Europas.* Goecke und Evers, Krefeld **1980**.
4. Aspöck, U.; Aspöck, H. *Dilar duelli* n. sp. -- eine neue Spezies der Familie Dilaridae aus Europa (Insecta: Neuropteroidea: Neuroptera). *Zeitschrift der Arbeitsgemeinschaft Österreichischer Entomologen* **1995**, 47: 49–54.
5. Aspöck, U., Liu, X.Y. & Aspöck, H. The Dilaridae of the Balkan Peninsula and of Anatolia (Insecta, Neuropterida, Neuroptera). *Deutsche Entomologische Zeitschrift* **2015**, 62, 123–135.
6. Badano, D. I Neurotteri (Neuropterida) della Liguria. In *Atti XXIII Congresso Nazionale Italiano di Entomologia,* 13–16 Giugno 2011, Genova. Abstract book; pp. 54.
7. Banks, N. Neuropteroid insects from Formosa. *Philippine Journal of Science* **1937**, 62: 255–291.
8. Bernardi Iori, A.; Kathirithamby, J.; Letardi, A.; Pantaleoni, R.A.; Principi, M.M. Neuropteroidea (Megaloptera, Raphidioptera, Planipennia), Mecoptera, Siphonaptera, Strepsiptera. *Checklist delle Specie della Fauna Italiana* **1995**, 62: 1–20.
9. Costa, A. Fauna del Regno di Napoli ossia enumerazione di tutti gli animali che abitano le diverse regioni di questo regno e le acque che le bagnano e descrizione de' nuovi o poco esattamente conosciuti con figure ricavate da originali viventi e dipinte al naturale. *Nevrotteri*. Napoli. **1860–1870**.
10. Devetak, D. Neuropteroidea. Megaloptera, Raphidioptera, Planipennia (Insecta). *Fauna Durmitora* **1991**, 4: 135–159.
11. Devetak, D. Megaloptera, Raphidioptera and Planipennia (Neuropteroidea, Insecta) of Croatia. *Znanstvena Revija* **1992a**, 4: 89–114.
12. Devetak, D. Present knowledge of the Megaloptera, Raphidioptera and Neuroptera of Yugoslavia (Insecta: Neuropteroidea). In *Current Research in Neuropterology*, Proceedings of the Fourth International Symposium on Neuropterology, Bagnères-de-Luchon, Haute-Garonne, France, 24–27 June 1991; M. Canard, H. Aspöck, M.W. Mansell, Eds.; Privately printed, Toulouse, France **1992b**; pp. 107–118.
13. Devetak D., Klokočovnik V., Rausch H., Janžekovič F. Fauna of the Neuropterida (Raphidioptera, Neuroptera) of the Protected Area Jasen, Macedonia: a summer flash. *Turkish Journal of Zoology* **2015**, 39: 15–27.
14. Díaz-Aranda, L.M., Monserrat, V.J. Contribución al conocimiento de los neurópteros de Jaén (Insecta, Neuroptera). *Boletín de la Asociación Española de Entomología* **1988a**, 12: 111-123.
15. Díaz-Aranda, L.M., Monserrat, V.J. Contribución al conocimiento de los neurópteros de Teruel (Insecta, Neuropteroidea). *Boletín de la Asociación Española de Entomología* **1988b**, 12: 215–231.
16. Dobosz, R.; Popov, A. New data about the distribution of Neuropterida in Bulgaria and Romania. *Rocznik Muzeum Górnoslaskiego w Bytomiu, Entomologia [=Annals of the Upper Silesian Museum in Bytom, Entomology]* **2018**, 27: 1–39].
17. Ghilarov, M.S. Личинка *Dilar turcicus* Hag. и положение семейства Dilaridae в отряде сетчатокрылых (Planippenia) [=The larva of *Dilar turcicus* Hag. and the position of the family Dilaridae in the order Planipennia]. *Entomological Review [=Энтомологическое Обозрение; =Entomologicheskoe Obozrenie]* **1962**, 41: 402–416.
18. Ghosh, S.K. Neuroptera fauna of north-east India. *Records of the Zoological Survey of India, Miscellaneous Publications, Occasional Paper* **2000**, 184: 1–179.
19. Giacomino, M. *Dilar parthenopaeus* Costa, 1855, new to the French fauna (Corsica) (Neuroptera, Dilaridae, Dilarinae). *Bulletin de la Société Entomologique de France* **2016**, 121: 81–86.
20. Hagen, H.A. Die Neuropteren Spaniens nach Ed. Pictet's Synopsis des Neuroptères d'Espagne. Genève 1865. 8. tab. 14 col. und Dr. Staudingers Mittheilungen. *Stettiner Entomologische Zeitung* **1866**, 27: 281–302.
21. Hollier, J.; Oswald, J.D. An annotated list of the type specimens of Megaloptera, Neuroptera and Raphidioptera in the collection of the Muséum d'histoire naturelle de Genève. *Revue Suisse de Zoologie* **2018**, 125: 249–261.
22. Klapálek, F. Über die von Herrn Prof. A. Hetschko in Korsika gesammelten Neuropteroiden nebst Bemerkungen über einige ungenügend bekannte Arten. *Wiener Entomologische Zeitung* **1917**, 36: 193–208.

23. Kuwayama, S. Studies on the Dilaridae of Japan. *Transactions of the Sapporo Natural History Society* **1921**, 8: 51–83.
24. Letardi, A. A web page on Italian Neuropterida. In *Neuropterology 2000*, Proceedings of the Seventh International Symposium on Neuropterology, Budapest, Hungary, 6–9 August 2000; Sziráki, G., Ed.; Acta Zoologica Academiae Scientiarum Hungaricae **2002**, 48: 155–158.
25. Letardi, A. *Dilar pumilus* Navás, 1903 and *Conwentzia pineticola* (Enderlein, 1905), two new lacewings for Continental Portugal (Neuroptera: Dilaridae, Coniopterygidae). *Arquivos Entomolóxicos* **2012**, 7: 71–72.
26. Li, D.; Liu, X.Y. New species of the pleasing lacewing genus *Dilar* Rambur, 1838 (Neuroptera, Dilaridae) from the Hengduan Mountains, southwestern China. *Journal of Asia-Pacific Entomology* **2020**, 23, 915–922.
27. Li, D., Aspöck, H., Aspöck, U. & Liu, X.Y. A review of the pleasing lacewing genus *Dilar* Rambur (Neuroptera, Dilaridae) from Central Asia. *Zootaxa* **2019**, 4671(1), 035–054.
28. Li, D., Aspöck, H., Aspöck, U. & Liu, X.Y. A review of the pleasing lacewing genus *Dilar* Rambur (Neuroptera, Dilaridae) from South Asia. *Zootaxa* **2020,** 4743(2): 200–216.
29. Marín, F. Las comunidades de neuropterós de la provincia de Albacete (Insecta: Neuropteroidea). *Al-Basit, Estudios Albacetenses* **1994**, 34: 247–304.
30. Marín, F.; Monserrat, V.J. Contribución al conocimiento de los neurópteros de Soria (Insecta, Neuropteroidea). *Boletín de la Asociación Española de Entomología* **1990**, 14: 219–230.
31. Marín, F.; Monserrat, V.J. Contribución al conocimiento de los neurópteros de Zaragoza (Insecta, Neuropteroidea). *Zapateri, Revista Aragonesa de Entomología* **1995**, 5: 109–126.
32. Martins, C.C., Flint, O.S.Jr. & Liu, X.Y. Notes on the pleasing lacewing genus *Dilar* Rambur, 1838 (Neuroptera, Dilaridae), with description of a new species from Vietnam. *Zootaxa* **2018**, 4425(1), 193–200.
33. Mirmoayedi, A.; Yassayie, A. *Dilar golestani* sp. n. (Neuroptera: Dilaridae) from Iran. *Journal of the Entomological Society of Iran* **1999**, 21, 15–18: 9.
34. Monserrat, V.J. Sobre los Neurópteros ibéricos (IV) (Neur.). *Boletín de la Asociación Española de Entomología* **1986**, 10: 95–105.
35. Monserrat V.J. Revision de la obra de L. Navás, I: El genero *Dilar* Rambur, 1842 (Neuropteroidea, Planipennia: Dilaridae). *Neuroptera International* **1988a**, 5, 13–23.
36. Monserrat, V.J. Revisión de los diláridos ibéricos (Neuropteroidea, Planipennia: Dilaridae). *EOS: Revista Española de Entomología* **1988b**, 64: 175–205.
37. Monserrat, V.J. Algunos Neuropteros del Museo de Basilea (Insecta, Neuropteroidea, Planipennia). *Entomologica Basiliensia* **1989**, 13, 417–428.
38. Monserrat, V.J. Nuevos datos sobre algunas pequeñas familias de neurópteros (Insecta: Neuroptera: Nevrorthidae, Osmylidae, Sisyridae, Dilaridae). *Heteropterus: Revista de Entomologia* **2005**, 5, 1–26.
39. Monserrat, V.J. Los Neurópteros (Neuroptera). Pp. 282-309 in Díaz Ruano, F.; Tierno de Figueroa, J.M.; Tinaut Ranera, J.A. *Los insectos de Sierra Nevada: 200 años de historia* **2013**, 1304 pp.
40. Monserrat, V.J. Los dilaridos de la península Ibérica (Insecta, Neuropterida, Neuroptera, Dilaridae). *Heteropterus: Revista de Entomología* **2014**, 14: 187–214.
41. Monserrat, V.J.; Díaz-Aranda, L.M. Contribución al conocimiento de los neurópteros de Castellón (Insecta, Neuropteroidea, Planipennia). *Mediterránea (Biologicos)* **1988**, 10: 87–95.
42. Morton, K.J. A collecting trip to the Camargue and the Sierra Albarracin. *Entomologist* **1912**, 45: 109–114.
43. Nakahara, W. A new *Dilar* species from Japan (Neur. Plan.). *Entomological News, Philadelphia* **1914**, 25: 297–298.
44. Nakahara, W. Formosan Neuroptera collected by the late Dr. T. Kano. *Kontyû* **1955a**, 23: 6–12.
45. Nakahara, W. The Dilaridae of Japan and Formosa (Neuroptera). *Kontyû* **1955b**, 23: 133–142.
46. Navás, L. Diláridos de España. *Memorias de la Real Academia de Ciencias y Artes de Barcelona* **1903**, (3)4: 373–381.
47. Navás, L. Monografía de la familia de los Diláridos (Ins. Neur.). *Memorias de la Real Academia de Ciencias y Artes de Barcelona* **1909**, 7, 619–671.
48. Navás, L. Quelques Nevroptères de la Sibérie méridionale-orientale. *Revue Russe d'Entomologie [=Русское Энтомологическое Обозрение; =Russkoe Entomologicheskoe Obozrenie]* **1912**, 12: 414–422.
49. Navás, L. Family Dilaridae. Neuroptera. *Genera Insectorum* **1914**, 156: 1–14.
50. Navás, L. Les dilarides (Ins. Névroptères) du Muséum National de Paris. *Bulletin du Muséum National d'Histoire Naturelle, Paris* **1925**, (1) 31: 188–189.
51. Navás, L. Insectos exóticos Neurópteros y afines. *Brotéria (Zoológica)* **1926**, 23: 79–93.
52. Navás, L. Insectos recogidos en España por el doctor F. Haas. *Boletín de la Sociedad Entomologica de España* **1927**, 10: 121–124.

53. Oswald, J.D. Annotated catalogue of the Dilaridae (Insecta: Neuroptera) of the World. *Tijdschrift voor Entomologie* **1998**, 141, 115–128.
54. Oswald, J.D.; Schiff, N.M. A new species of the genus *Dilar* Rambur (Neuroptera: Dilaridae) from Borneo. *Proceedings of the Entomological Society of Washington* **2001**, 103, 74–80.
55. Pantaleoni, R.A.; Letardi, A. A remarkable brachypterous female of Dilaridae (*Dilar parthenopaeus* Costa?) (Neuroptera). Proceedings of the XX International Congress of Entomology. Firenze, Italy, August 25–31, **1996**: 62.
56. Pongrácz, S. Újabb adatok Magyarország Neuroptera-faunájához. *Rovartani Lapok* **1913**, 20: 175–186.
57. Popov, A. What do we know on the distribution of Neuropteroidea in Bulgaria. *Priroda* **1964**, 13: 68–72.
58. Popov, A. Raphidiopteren und Neuropteren aus Bulgarien in den Sammlungen des Nationalmuseums in Prag. *Historia Naturalis Bulgarica* **1993**, 4: 16–28.
59. Popov, A. The snakeflies and the lacewing insects (Insecta: Raphidioptera and Neuroptera) of the Kresna Gorge (SW Bulgaria). In: *Biodiversity of Kresna Gorge (SW Bulgaria)*; Beron P., Ed.; National Museum of Natural History, Sofia **2001**, 131–143.
60. Popov, A. Zoogeographical analysis of Neuroptera in Bulgaria. *Acta Zoologica Academiae Scientiarum Hungaricae* **2002**, 48: 271–280.
61. Popov, A. List of Raphidioptera, Neuroptera and Mecoptera published from the Eastern Rhodopes (Bulgaria and Greece). In: *Biodiversity of Eastern Rhodopes (Bulgaria and Greece);* Beron P., Popov A., Eds.; National Museum of Natural History, Sofia **2004**, 371–378.
62. Popov, A. Distribution of the families of Neuroptera with low species diversity in Bulgaria. Proceedings of the 9th International Symposium on Neuropterology, Ferrara 20–23 June 2005; Pantaleoni R.A., Letardi A., Corazza C., Eds.; Annali del Museo Civico di Storia Naturale di Ferrara **2007**, 8: 117–130.
63. Tjeder, B. A contribution to the phylogeny of the Dilaridae and the Raphidiidae (Neuroptera). *Opuscula Entomologica* **1937**, 2: 138–148.
64. Vidal y López, M. Catalogo provisional de neuropteros de la region Levantina. *Graellsia* 1943, 1(6): 13–24.
65. Whittington, A.E. Resources in Scottish Neuropterology. In: *Neuropterology 2000*. Proceedings of the Seventh International Symposium on Neuropterology, Budapest, Hungary, 6–9 August 2000; Sziráki, G., Ed.; Acta Zoologica Academiae Scientiarum Hungaricae 48: 371–387.
66. Zakharenko, A.V. Setchatokrylye (Neuroptera) fauny SSSR. II. Semeistva Dilaridae, Berothidae i Sisyridae [= Neuroptera of the fauna of the USSR. II. Fam. Dilaridae, Berothidae and Sisyridae]. *Entomologicheskoe Obozrenie* **1988**, 67: 763–768. [In Russian]
67. Zakharenko, A.V.; Krivokhatsky, V.A. Setchatokrylye (Neuroptera) evropeiskoi chasti byvshego SSSR [= Neuroptera from the European part of the former USSR]. Izvestiya Kharkovskogo Entomologicheskogo Obshchestva [= Kharkov Entomological Society Gazette] **1993**, 1: 34–83. [In Russian]
68. Zelený, J. Neuroptera, Megaloptera und Mecoptera aus Bulgarien. *Sborník Faunistickych Prací Entomologického Oddelení N rodního Musea v Praze [=Acta Faunistica Entomologica Musei Nationalis Pragae]* **1971**, 14: 153–163.
69. Zhang, W., Liu, X.Y., Aspöck, H. & Aspöck, U. Revision of Chinese Dilaridae (Insecta: Neuroptera) (Part I): Species of the genus *Dilar* Rambur from northern China. *Zootaxa* **2014a**, 3753, 10–24.
70. Zhang, W., Liu, X.Y., Aspöck, H. & Aspöck, U. Revision of Chinese Dilaridae (Insecta: Neuroptera) (Part II): Species of the genus *Dilar* Rambur from Tibet. *Zootaxa* **2014b**, 3878, 551–562.
71. Zhang, W., Liu, X.Y., Aspöck, H., Winterton, S.L. & Aspöck, U. Species of the pleasing lacewing genus *Dilar* Rambur (Neuroptera, Dilaridae) from islands of East Asia. *Deutsche Entomologische Zeitschrift* **2014c**, 61, 141–153.
72. Zhang, W., Liu, X.Y., Aspöck, H. & Aspöck, U. Revision of Chinese Dilaridae (Insecta: Neuroptera) (Part III): Species of the genus *Dilar* Rambur from the southern part of mainland China. *Zootaxa* **2015**, 3974, 451–494.
73. Zhang, W., Liu, X.Y., Winterton, S.L., Aspöck, H. & Aspöck, U. A review of the pleasing lacewing genus *Dilar* Rambur (Neuroptera, Dilaridae) from Southeast Asia. *Zootaxa* **2016**, 4105, 124–144.
